# The appropriate expression and coordination of glycolate oxidase and catalase are vital to the successful construction of the photorespiratory metabolic pathway

**DOI:** 10.3389/fpls.2022.999757

**Published:** 2022-10-27

**Authors:** Zhen Yao, Zelai Rao, ShuWang Hou, Changwei Tian, Chun-Yan Liu, Xiulan Yang, Guicai Zhu

**Affiliations:** ^1^ College of Horticulture and Gardening, Yangtze University, Jingzhou, China; ^2^ School of Finance and Economics, Jimei University, Xiamen, China; ^3^ Department of Medicine, Yangtze University, Jingzhou, China

**Keywords:** metabolic branches of photorespiration, glycolate oxidase, catalase, fusion expression, potato

## Abstract

Photorespiration has emerged as a hotspot in the evolution of photosynthesis owing to the energy loss during the process. To ensure the physiological functions of photorespiration such as light protection, H_2_O_2_ signaling, and stress resistance, separate the photorespiration glycolic acid flow, and minimize photorespiration loss, a balance must be maintained during the construction of photorespiratory metabolic branch. In this study, glycolate oxidase (GLO) and catalase (CAT) were introduced into potato (*Solanum tuberosum*) chloroplasts through the expression of fusion protein. Through the examination of phenotypic characteristics, photosynthesis, anatomical structure, and enzyme activity, the efficiency of the photorespiration pathway was demonstrated. The results showed that certain transgenic lines plants had shorter plant height and deformed leaves and tubers in addition to the favorable photosynthetic phenotypes of thicker leaves and larger and denser mesophyll cells. By Diaminobenzidine (DAB) staining analysis of the leaves, the intermediate H_2_O_2_ could not be decomposed in time to cause biomass decline and malformation, and the excessive glycolate shunt formed by the overexpression of the fusion protein affected other important physiological activities. Hence, the appropriate and coordinated expression of glycolate oxidase and catalase is essential for the establishment of photorespiration pathways in chloroplasts.

## Introduction

Potato is the staple food of many developing countries and is the only one of the four major food crops in the world that uses nutrition for reproduction. Scientists have gradually realized that the potential of potatoes in increasing production is much higher than that of rice, wheat, and corn. Research on potatoes breeding and stress resistance was becoming increasingly important to cope with the growing global demand for food and energy (Gervais et al., 2021; Gutiérrez-Valencia and Slotte, 2022). As early as 2011, whole-genome sequencing was completed on potatoes, and studies of their genomic evolution and genetic diversity have further reduced barriers to potato breeding (Potato Genome Sequencing Consortium et al., 2011; Tang et al., 2022). Compared to sexually reproducing species, potatoes can be conveniently and stably preserved after genetic improvement. And they have been intensively studied in terms of drought resistance, disease resistance, processing quality, and high yield (Hameed et al., 2018). In addition, potato tubers are the main harvested organs and consist mainly of photosynthetic products. The increase in plant biomass can be more efficiently manifested in the product organs without being limited by the conversion efficiency of the vegetative reproductive process (Dahal et al., 2019). Therefore, potato is an excellent plant material for photosynthesis research.

Photorespiration is a biochemical process that occurs in all photosynthetic carbon-fixed cells exposed to light. It lowers the overall efficiency of photosynthesis as a side effect of energy loss during photosynthesis (Bauwe et al., 2010; Maurino and Peterhansel, 2010). However, photorespiration also affects many physiological activities such as photoprotection, H_2_O_2_ signaling, nitrate assimilation, and biological and abiotic stress resistance (Betti et al., 2016; Eisenhut et al., 2019). Previous studies have confirmed the importance of photorespiration metabolic pathway in *Arabidopsis* (Kebeish et al., 2007; Maier et al., 2012), rice (Shen et al., 2019) and potato (Nölke et al., 2014) in boosting biomass and minimizing the harm caused by CO_2_ deficits and surplus energy under abiotic stress such as high temperature and drought conditions. Reducing the loss of carbon, nitrogen, and energy by diverting glycolic acid produced by RuBP (1,5-ribulose diphosphate) oxidation; avoiding the toxicity of metabolizing photorespiration intermediates; and enhancing photosynthesis and biomass production are the common points of the three effective photorespiration branches (Maier et al., 2012; Dalal et al., 2015; Shen et al., 2019; Yao et al., 2020).

Kebeish et al. (2007), introduced five genes from bacteria metabolizing glycolate into *Arabidopsis*, resulting in transformed plants capable of directly converting the glycolate of chloroplasts into glycerate. The above research is generally referred to as branch. Branch 1 reduces the release of ammonia, recovers 75% of the glycolic acid lost during photorespiration, releases the glycolic acid produced by photorespiration into CO_2_, and transfers it to chloroplasts utilizing three enzymes from bacterial glycolic acid metabolism (EcGDH, EcGCL, EcTSR). Using newly added enzymes and primary enzymes, branch 2 glycol acid is entirely oxidized to CO_2_ in the chloroplast, raising the intercellular CO_2_ content in the chloroplast (Maier et al., 2012). Three rice autogenous enzymes, OsGLO3, OsOXO3, and OsCATC, convert glycolic acid in branch 3 into CO_2_ in the chloroplast, raising the CO_2_ content and lowering the emission of ammonia (Shen et al., 2019). To alter the photosynthesis in potato, a fusion protein is created from the three glycolate dehydrogenase subunits of *E. coli* (Nölke et al., 2014). This protein successfully produces a photorespiration branch in potato chloroplasts and boosts the biomass of potato tubers.

Enzyme activity cannot be appropriately coordinated in some plants with a well-established photorespiration pathway (Maier et al., 2012). The main reason for this phenomenon is mostly due to the necessity to simultaneously introduce several genes to build a metabolic branch. Expression box accumulation, progressive transformation, hybridization, and other techniques are the main techniques employed. To ensure that the introduced genes are expressed normally, it is necessary to successfully construct a metabolic branch, followed by accurate localization and coordination of expression. Photorespiration is also required in plants with slow RuBP oxidation rate, which cannot be entirely suppressed (Peterhansel and Maurino, 2011). An appropriate shunt is a prerequisite for the success of all metabolic pathways. Moreover, many metabolic products are harmful to organelles. It matters more whether they can be successfully broken down by the added enzymes or by their own metabolic system.

*Oryza sativa* glycolate oxidase 3 (OsGLO3) can efficiently convert glycolic acid to glyoxylic acid, and its enzymatic activity is intermediate among the four isozymes, producing the metabolite hydrogen peroxide (Zhang et al., 2012). Glycolate is the first product produced in the chloroplast *via* photorespiration processes, and is transported out of the chloroplast by the plastid glycolate/glycerate translocator and into other organelles (Shim et al., 2020). Glycolic acid is the focus of the photorespiration shunt, and *Oryza sativa* catalase C (OsCATC) can remove any H_2_O_2_ resulting from the reaction (Zhang et al., 2016; Bao et al., 2021). These two genes have been shown to play an important role in the construction of photorespiration pathway in rice (Shen et al., 2019).

To better coordinate the expression of these two enzymes, their expressions are coordinated by fusion expression. In the present study, two enzymes from plants were introduced into potato chloroplasts: OsGLO3, a subtype of glycolate oxidase, and OsCATC a subtype of catalase, and appropriate isozymes are selected. By enzyme expression quantity and phenotype correspondence analysis, we confirmed the photorespiration metabolic branch segregated glycolic acid by examining the phenotypic characteristics, photosynthesis, anatomical structure, and enzyme activity of transgenic potato plants. Glycolate oxidase (GLO) and catalase (CAT) can be properly expressed and coordinated to provide a positive transgenic phenotype that allows for the rational diversion of glycolate and the elimination of hydrogen peroxide.

## Materials and methods

### Transformation vector construction and potato transformation plant acquisition

The coding sequences of OsCAT2 (AK062174) and OsGLO3 (AK068638) were amplified from rice cDNA. The coding regions of the two genes were fused through a 3-junction encoding 12 glycine and 3 serine (Gly4Ser), and the encoded N-terminal was connected to the potato *rbcS* chloroplast localization signal peptide. The expression of the fusion protein was initiated by the tobacco mosaic virus 35S promoter. Potato (cultivar E-potato 3 (E3)) was transformed by Agrobacterium-mediated method, and the transgenic lines were screened through DNA-level and protein-level expression analyses.

Uniformly sized tubers were selected as seed tubers in the first generation. Among the transgenic lines with stable expression, four lines with stable phenotypes were selected (B11, B14, B17, and B27). Each plant line was randomly planted in six pots, with one plant in each pot, making a total of 30 pots. The time from planting to harvest was 20 weeks for short-day conditions and 16–20 weeks for late-stage tuber bulking conditions (In southern China, potatoes are mainly planted in spring or autumn, and it generally takes five months from planting to harvesting in the field.). At the late stage of tuber expansion, the plant height was counted. The second top leaf in full expedition was selected to measure the photosynthetic parameters using a Li-6400 portable photosynthesis system (LICOR Biosciences, Lincoln, NE, USA) to obtain the net photosynthetic rate (*Pn*) under the condition of 400 µmol/mol [CO_2_] on a sunny morning at approximately 09:00 to 12:00 and 15:00 to 17:00 pm (It does exist that some plants had decreased photosynthesis at 15:00-17:00. The present study measured the net photosynthetic rate of potato leaves at 9:00-17:00 in a pre experiment. For formal experiments, 9:00-12:00 and 15:00-17:00 were relatively stable. Potato is a cold and cool crop and the temperature in the afternoon of the growing season is not particularly high, so it is reasonable to measure it at 15:00-17:00.). The third top leaf in full expedition was selected to measure the leaf shape and leaf area using a Wanshen LA-S versatile plant image analyzer (LA-S, Wanshen Detection technologies, Zhejiang, China).

### Enzyme activity analysis

GLO activity (phenylhydrazine hydrochloride method) was assayed with some modifications (Xu et al., 2009), and the amount of glyoxylic acid produced was determined at 324 nm wavelength for 1 min. Meanwhile, the method used to determine the H_2_O_2_ consumption at 520 nm wavelength for 1 min was referred to in the determination of GLO activity by POD method (Wang et al., 1999). The change of hydrogen peroxide concentration was detected at 520nm to evaluate the activity of GLO enzyme. Catalase (CAT) activity was determined based on the consumption of H_2_O_2_ (extinction coefficient of 43.6 M^-1^cm^-1^) at 240 nm for 1 min (Abei, 1984).

### Analysis of leaf anatomical structure

In a natural environment (13°C-21°C, under short-day, 900Lux -1200Lux, peat soil planting), each plant line (test tube plantlet) has five mature healthy leaves with the same growth status and size from different individual plants, and approximately three leaf tissues with a size of 5 mm × 8 mm were cut at the obvious midrib of the leaflet and fixed with FAA fixative. After the materials were embedded, they were continuously sectioned with a paraffin slicer with a section thickness of 20 µ M, and Toluidine Blue O (TBO) staining and neutral gum sealing were performed. Images were taken with an Olympus Dp71 micrographic system, and the indicators were observed using Image-ProPlus 6.3. Software. 30 replicates were set for each treatment. SPSS 20.0 software was used for analysis. ANOVA using Tukey-Kramer posthoc test (*P* < 0.05).

### *In situ* localization of H_2_O_2_ in leaves

Referring to the method of Daudi and O’Brien (2012), five plants were selected from each line, and three leaves with the same leaf position and leaf conditions were selected from each plant. They were quickly inserted into the shading DAB staining solution, which composed of 1 mg ml^-1^ DAB solution at pH 3, Tween 20 (0.05% v/v), and 200 mM Na_2_HPO_4_. They were then shaken in the laboratory at 100 rpm for 4 h and then decolorized with a bleaching solution (ethanol: acetic acid: glycerol =3:1:1) in a 95°C thermostatic water bath for 20 min. Photographs were obtained after decolorization.

## Results

### Two phenotypes of transgenic lines

A series of transgenic lines were obtained by introducing glycolate oxidase and catalase into potato chloroplasts. The plant height, leaf shape, leaf color, and tuber external morphology of the transgenic lines with normal phenotypes (B11 and B14) were identical to those of the wild type.

**Figure 1:**
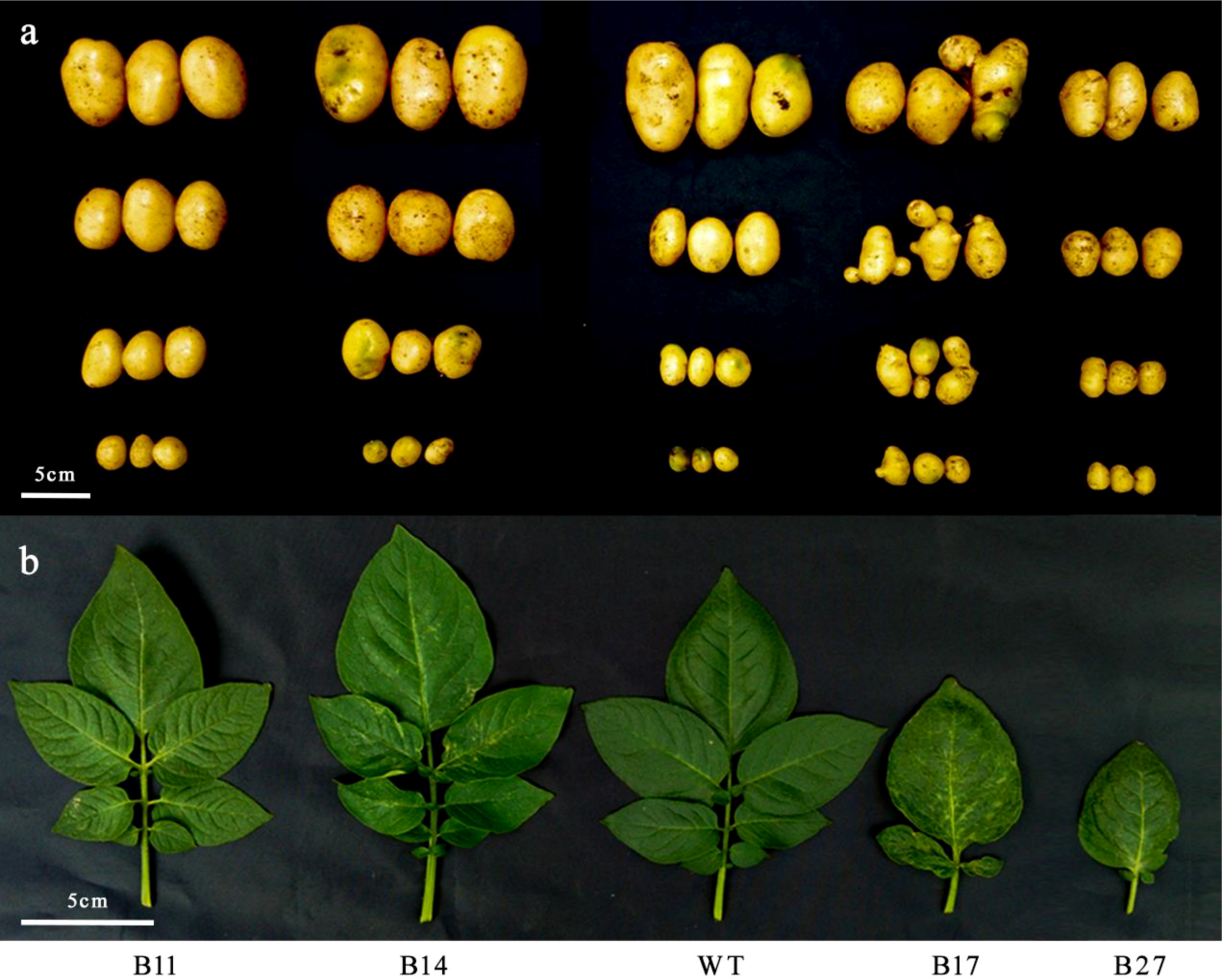
Phenotype of the second-generation plants of tuber propagation. Comparison of tuber morphology **(A)** and leaf **(B)** between transgenic and wild type.

B17 and B27, two transgenic lines, simultaneously displayed an abnormal phenotype. The plants were shorter when they were test tube seedlings. The leaves of the plants were twisted, thicker, brittle, darker, and connected as they reached the tuber expansion stage. The effective leaf number of strain B17 was 19.9 ± 10.70 (mean and standard error), the effective leaf number of strain B27 was 27.75 ± 5.73, and the effective leaf number of wild type was 40.13 ± 3.40. The effective leaf number of these two strains was significantly different from that of wild type (*P* < 0.05). The number of effective leaves decreased by 31% and 50%, respectively compared to that of the wild type (**Figure 1**). Multiple large and small tubers grew together in the deformed and distorted tubers. These traits were observed upon subsequent multigenerational vegetative propagation.

### Leaf thickening of transgenic plants with phenotypic abnormalities

**Figure 2:**
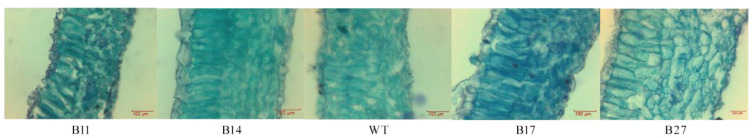
Paraffin section observation of transgenic plants. The mesophyll cells in the leaves of B27 and B17 grew thicker, larger, and more densely packed, whereas the mesophyll cells in the leaves of B11 and B14 shrank.

**Figure 3:**
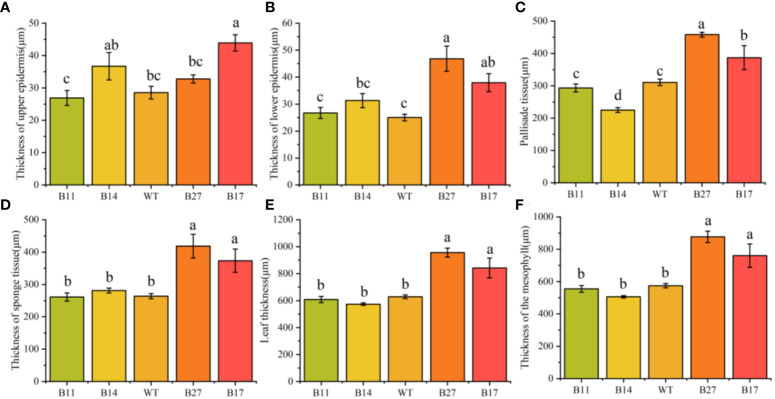
Statistical analysis of thickness (n=30) of upper epidermis, lower epidermis, palisade tissue, sponge tissue, leaf and mesophyll cell of asexually propagated second generation transgenic plants. Data are means ± SE of 30 biological replicates. Different letters indicate statistically significant differences between genotypes as determined by ANOVA using Tukey-Kramer posthoc test (P < 0.05). **(A)** Thickness of upper leaf epidermis of transgenic and wild type. **(B)** Thickness of lower leaf epidermis of transgenic and wild type. **(C)** Thickness of palisade tissue of transgenic and wild type. **(D)** Thickness of sponge tissue of transgenic and wild type. **(E)** Thickness of leaf of transgenic and wild type. **(F)** Thickness of mesophyll of transgenic and wild type.

Transgenic leaf lines were paraffin sectioned and examined under a microscope (**Figure 2**). The mesophyll cells in the leaves of B27 and B17 grew thicker, larger, and more densely packed, according to the paraffin sections, whereas the mesophyll cells in the leaves of B11 and B14 shrank. Quantitative analysis showed that the leaf thickness and mesophyll cell thickness of transgenic plants B11 and B14 were lower than those of wild-type plants, and the thickness of B14 palisade tissue was significantly lower than that of the wild type by 28% (*P* < 0.05 **Figure 3**).

Our transgenic potato leaves had two distinct phenotypes, indicating that different plants express different forms of the fusion protein. We hypothesize that the overexpression of the fusion protein causes the B27 and B17 leaves to thicken, affects physiological processes, and decreases biomass.

### Analysis of net photosynthetic rate in potato plants

**Figure 4:**
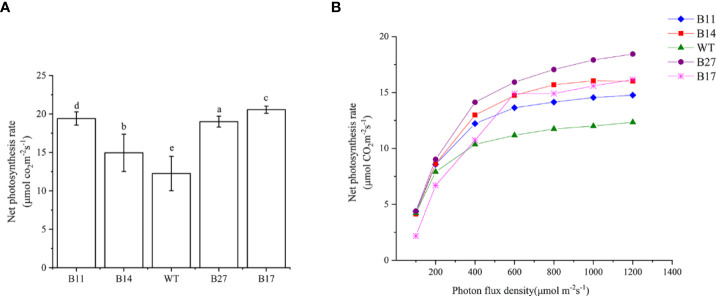
Net photosynthetic rate of the transgenic plants and light response curves of transgenics produced by asexual propagation for two generations. **(A)** Net photosynthetic rate of the transgenic plants. At 700 μ mol m^-2^s^-1^ light intensity, temperature 30°C, normal atmospheric conditions (CO_2_ concentration of approximately 400 μ mol) was determined. Data are means ± SE of 30 biological replicates. Different letters indicate statistically significant differences between genotypes as determined by ANOVA using Tukey-Kramer posthoc test (P < 0.05). **(B)** Light response curves of transgenics produced by asexual propagation for two generations. At a temperature of 30°C, normal atmospheric conditions (CO2 concentration of approximately 400 μ mol) were used.

The net photosynthetic rate of the T0 transgenic plants during the expansion stage was measured, and the results are shown in **Figure 4A**. Under 1000 μ mol m^-2^s^-1^ light intensity, 30 °C temperature, and normal atmospheric conditions, the net photosynthetic rate of the wild-type potato was 12.26 ± 2.24μmol CO_2_ m^-2^s^-1^. The net photosynthetic rates of transgenic lines B11, B14, B27, and B17 were 19.41 ± 0.86, 14.95 ± 2.42, 19.00 ± 0.71, and 20.55 ± 0.47 μ mol CO_2_ m^-2^s^-1^, respectively, and they increased by 58%, 22%, 55%, and 68% (*P* < 0.05 **Figure 3**), respectively compared with the wild type.

Photoresponse curves of GLO-CAT transgenic plants were established, and the results are shown in **Figure 4B**. The photosynthetic rate of B17 transgenic plants was lower than that of wild–type plant in the linear region (light intensity less than 200 mol CO_2_ m^-2^s^-1^), and there was no difference between B11, B14, or B27 transgenic plants and wild-type plants. However, in the transition region (light intensity greater than 200 mol CO_2_ m^-2^s^-1^), the net photosynthetic rate of all transgenic lines was higher than that of wild type, and the difference became increasingly obvious with the increase in light intensity. The light saturation rate Amax of all transgenic lines was much higher than that of the wild type when the light saturation point LSP was achieved. This demonstrates that the photorespiration branch plays a role in recovering some of the photorespiration CO_2_ and the transgenic plants are less constrained by intercellular CO_2_ concentration than wild-type plants.

### The GLO and CAT activities of the fusion protein in transgenic plant

**Figure 5:**
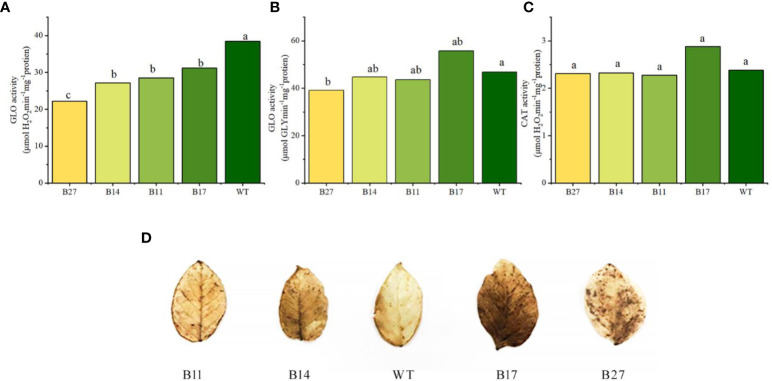
Analysis of GLO and CAT enzyme activities in transgenic plants and intravital *in situ* DAB staining of H_2_O_2_ in leaves of transgenic plants. Different letters indicate statistically significant differences between genotypes as determined by ANOVA using Tukey-Kramer posthoc test (P < 0.05). Data sets without letters indicate no statistical differences. **(A)** GLO activity measured by POD method. Data are means ± SE of 30 biological replicates. **(B)** GLO activity measured by phenylhydrazine hydrochloride method. Data are means ± SE of 30 biological replicates. **(C)** CAT activity. Data are means ± SE of 30 biological replicates. **(D)** Intravital *in situ* DAB staining of H_2_O_2_ in leaves of transgenic plants.

GLO and CAT activities during potato tuber expansion were measured. Compared to the wild type, the GLO activity values — transgenic lines B11 and B14 grew slightly, B17 increased by 26%,. However, no significant difference was observed, and B27 declined slightly (*P* < 0.05). according to the results of the phenylhydrazine hydrochloride method (glyoxylate GLY determination), as shown in **Figures 5A–C**. B11, B14, B27, and B17 were considerably lower than wide-type plant (*P* < 0.05), according to the POD method (used to measure H_2_O_2_). According to the CAT activity values, B11, B14, and B27 were comparable to wide-type plant, but that of B17 was 32% greater than that of wide-type plant.

It is clear from the results above that the POD method was used to gauge the enzyme activity of GLO in relation to the H_2_O_2_ generated during the reaction. The fusion protein simultaneously expressed CAT enzyme activity. Low GLO enzyme activity is caused by the CAT activity of fusion protein in the quick breakdown of H_2_O_2_, which occurs during the determination process. The above information demonstrates that GLO and CAT enzyme activities of the fusion protein were successfully expressed in transgenic plants.

### *In situ* localization revealed that H_2_O_2_ cannot be decomposed in time

The breakdown of glycolic acid by GLO results in a significant amount of H_2_O_2_ being produced in the photorespiration metabolic transformation branch of potato. However, as CAT enzyme activity in transgenic plants is not significantly increased, it may not be possible to completely or timely remove the H_2_O_2_ produced by the photorespiration branch, and this affect the development of transgenic plants and result in abnormal phenotypes. The H_2_O_2_ levels in the leaves of wild-type and transgenic lines at the tuber expansion stage were examined using DAB staining to verify this idea. The color of the transgenic plant leaves was darker than those of wild-type plants (**Figure 5D** displays the outcomes). The H_2_O_2_ content in the leaves of transgenic plants was higher than that of wild-type plants; it was higher in B27 and B17 plants with abnormal phenotypes and slightly lower in B11 and B14 plants with normal phenotypes.

## Discussion

Research on the photorespiratory metabolic branch has taken more than 10 years from its establishment to the present, with progress successively on *Arabidopsis*, rice, potato. However, there have been fewer successful cases in recent years, which may be limited by plant transformation conditions, selection of genes, restriction of multigene introduction coordination and so on. Besides, CO2-concentrating mechanism establish in C3 plant chloroplasts remains elusive (Rottet et al., 2021). The search for suitable glycolate shunts and coordinated expression between foreign genes represents a new breakthrough point to improve the efficiency of branched construction.

Positive results were observed when the GLO-CAT fusion protein was added to the photorespiration pathway built by the potato chloroplasts. Firstly, the plant height, leaf area, number of effective leaves, and potato weight of transgenic plant B14 increased significantly, as did the photosynthetic rate, supporting the positive effects of the photorespiration branch put in place by previous researchers (Maier et al., 2012; Dalal et al., 2015; Shen et al., 2019; Shi and Bloom, 2021). Secondly, transgenic plants B17 and B27 developed brittle, thicker, and darker leaves but the photosynthetic rate did not decline significantly. Shen et al. (2019) and Busch (2020) pointed out that the greening of rice leaves and the enlargement of leaf cells in the branch were attributed to the diversion of glycolic acid in the photorespiration branch, which reduced photorespiration and the loss of chlorophyll-related N. This suggests that B27 and B17 still benefit from the production of fusion protein. Thirdly, while *Arabidopsis* only exhibits a branch advantage under poor light and brief sunshine settings, transgenic plants B11, B14, B27, and B17 have a growth advantage under high light conditions (Peterhansel and Maurino, 2011). Although, strong light results in surplus ATP and reduction capacity, photosynthesis is often constrained by CO_2_ concentration (Shen et al., 2019). Light response curve of our potato and higher light saturation rate Amax and light saturation point LSP were observed, indicating that the expression of the fusion protein increased the intercellular CO_2_ concentration of the chloroplasts of transgenic plants.

Potato mosaic virus is the primary source of leaf deformation (Cuellar et al., 2015); however, the leaf deformity in this study is distinct from that caused by the virus. The manifestations caused by virus disease are different from the phenotypes observed in this study, which are dwarf diseased strains, small and severely shrunken leaves, upward bending of leaf margins, and dark color of leaf flesh. There are no comparable reports on tuber malformations. By consulting the research literature on potato leaf malformation and comparing the phenotypes, it is found that the phenotype caused by potato mosaic virus is the most important one related to this study. The vascular bundle necrosis caused by the virus impedes the transport of nutrients, resulting in the inhibition or accumulation of chlorophyll synthesis in leaves. The effect on chlorophyll is related to the metabolic transformation of chloroplasts in the present study. Moreover, the transformed wild-type was used as virus-free material, and there was no interference of virus disease.

It has been demonstrated that it is essential to regulate the activity of certain transgenic enzymes in chloroplasts (Maier et al., 2012). The phenotypes of B11 and B14 in this study were identical, and B14 biomass and potato weight increased dramatically, demonstrating that the GLO-CAT fusion protein was moderately expressed without affecting other physiological and metabolic functions. In the second phenotypic analysis, the photosynthetic rate, leaf tuber deformities, and B17 and B27 biomass decreased. We discussed three possibilities. (1) Because the expression level of the fusion protein was too high, GLO broke down glycolic acid to produce a large amount of H_2_O_2_ but CAT could not develop in the chloroplast on time to prevent it from producing too much H_2_O_2_; (2) The expression of the fusion protein was still not coordinated, and the CAT enzyme activity of the fusion protein was unstable and could not release the expected activity; (3) Glyoxylic acid did not break down and had negative effects, which may be related to GLO overexpression. The leaf deformity phenotype has been observed in studies of potato mismatch repair deficiency and somatic hybridization. The main manifestations of malformation are slightly grayish-green colored and deformed leaves, which are similar to characteristics of the phenotypes observed in the present study; however, there were still differences (Rakosy-Tican et al., 2019).

Several studies have reported that the micromolar concentrations of glyoxylic acid can significantly inhibit plant photosynthesis by inhibiting the activation of Rubisco (Chastain and Ogren, 1989; Campbell and Ogren, 1990; Lu et al., 2014), and it is important to introduce an appropriate amount of Rubisco (Keech et al., 2017). However, Kebeish overexpressed glycolate dehydrogenase in *Arabidopsis* chloroplasts resulted in glyoxylic acid accumulation, which improves photosynthesis and increases biomass.

A certain amount of glyoxylate that accumulates, undergoes transport between organelles and breakdown by other metabolic pathways. But if the scope of metabolism is exceeded, it may cause a bad phenotype, affecting photosynthesis.

According to a report, the overexpression of glycolate oxidase in *A. thaliana* chloroplasts causes H_2_O_2_ to build up, plants to develop more slowly, rosettes to turn yellow, and photosynthetic efficiency to diminish (Fahnenstich et al., 2008). Further verification confirmed that the first conjecture was most likely. DAB staining revealed that H_2_O_2_ accumulated in B27 and B17 leaves and only a small amount of H_2_O_2_ remained in B11 and B14, despite the fact that the two methods for assessing GLO activity confirmed that CAT was expressed in all transgenic lines. H_2_O_2_ can harm DNA, proteins, and lipids and may lead to tissue necrosis, according to certain researches (Barry, 2006). H_2_O_2_ inhibits the genes encoding photosynthetic components and directly destroys or inhibits the activities of enzymes involved in CO_2_ assimilation and energy metabolism (Vandenabeele et al., 2003; Scarpeci and Valle, 2008; Palatnik et al., 1999; Asada, 2006; Baxter et al., 2007), and this explains why B17 and B27 had decreased biomass, leaf tuber deformity, and photosynthetic rate. Our results are also consistent with the overexpression of glycolate oxidase and the accumulation of H_2_O_2_ in *A. thaliana* chloroplasts by Fahnenstich, which resulted in plant growth retardation, smaller and yellowing rosettes, and decreased photosynthetic performance (Fahnenstich et al., 2008). Therefore, we speculate that CAT expression in B17 and B27 fusion proteins is too low compared to GLO, and H_2_O_2_ produced by glycolic acid decomposition by GLO is not removed on time, resulting in a large amount of H_2_O_2_ accumulated in leaves and poisoning plants.

In conclusion, our findings demonstrate that when building photorespiration pathways in potato chloroplasts, it is crucial for glycolate oxidase and catalase to be properly and coordinately expressed. The photosynthetic efficiency of plants can be enhanced by the moderate expression of the fusion protein, which is directly reflected in the phenotype; that is, the biomass increases and the tuber yield increases. Future work will focus on testing the viability of this approach to increase the production of other crop varieties and introduce proper downstream genes, creating a more comprehensive photorespiration branch and other related tasks.

## Data availability statement

The raw data supporting the conclusions of this article will be made available by the authors, without undue reservation.

## Author contributions

GZ, ZY and XY conceived and designed the research. ZY and ZR conducted experiments and wrote the manuscript. SH and C-YL translated and polished the manuscript. CT and SH made the tables and pictures. All authors contributed to the article and approved the submitted version.

## Conflict of interest

The authors declare that the research was conducted in the absence of any commercial or financial relationships that could be construed as a potential conflict of interest.

## Publisher’s note

All claims expressed in this article are solely those of the authors and do not necessarily represent those of their affiliated organizations, or those of the publisher, the editors and the reviewers. Any product that may be evaluated in this article, or claim that may be made by its manufacturer, is not guaranteed or endorsed by the publisher.
