# Supplementary figures and images for: The appropriate expression and coordination of glycolate oxidase and catalase are vital to the successful construction of the photorespiratory metabolic pathway
Source: Front Plant Sci. 2022 Oct 27;13:999757. doi: 10.3389/fpls.2022.999757 (PMC9647076; doi:10.3389/fpls.2022.999757)

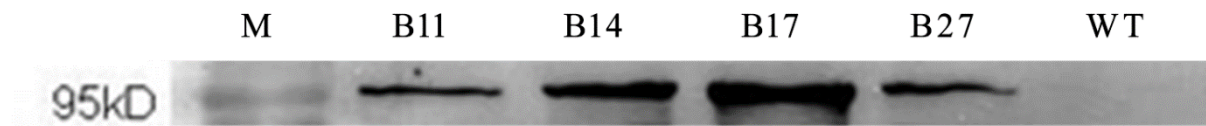

**Supplemental Fig.1** Western blot analysis of transgenic lines (his antibody)

Supplement: Supplementary file 1 [file Image_1.pdf]
